# Severe Liver Injury Secondary to COVID-19-Induced Rhabdomyolysis in McArdle Disease

**DOI:** 10.7759/cureus.34160

**Published:** 2023-01-24

**Authors:** Ana P Urena Neme, Carol Fernandez Hazim, Gustavo Duarte, Michael Victoria Guerrero, Miguel A Rodriguez Guerra

**Affiliations:** 1 Cardiology, Medicina Cardiovascular Asociada, Santo Domingo, DOM; 2 Medicine, Montefiore Wakefield Hospital, Bronx, USA; 3 Medicine, Jacobi Medical Center, Bronx, USA; 4 Medicine, Instituto Tecnologico de Santo Domingo, Santo Domingo, DOM; 5 Medicine, Montefiore Medical Center, Albert Einstein College of Medicine, Bronx, USA

**Keywords:** aki outcome, sars covid-19, covid 19, rhabdomyolysis, acute liver failure (alf), mcardle disease, glycogen storage disorder

## Abstract

Severe liver injury is an uncommon condition caused by non-traumatic rhabdomyolysis. This rare correlation is more commonly seen in the aspartate aminotransferase (AST) than in the alanine transaminase (ALT) level elevation. We report a case of a 27-year-old male with a history of McArdle disease who presented with generalized muscle aches associated with dark urine. His workup showed SARS-CoV-2 positive, severe rhabdomyolysis (creatinine kinase [CK] > 40000 U/L) and acute kidney injury (AKI) followed by severe liver injury (AST/ALT: 2122/383 U/L). He was started on aggressive intravenous hydration. After multiple boluses, he became overloaded, fluids were re-adjusted and continued, his renal function, CK, and liver enzymes improved, and the patient was discharged; during his visit at the post-discharge, the patient was asymptomatic and no clinical or laboratory abnormalities were found. The glycogen storage diseases are challenging, but prompt and accurate assessment is determinant in recognizing potential life-threatening complications of SARS-CoV-2. The failure to identify complicated rhabdomyolysis could lead to the patient's rapid deterioration, ending in multiorgan failure.

## Introduction

Myophosphorylase (muscle phosphorylase) enzyme deficiency, an uncommon autosomal recessive glycogen storage disorder (GSD) also known as McArdle disease, was first reported in 1951 [1,2]. This condition affects 1 in 100,000 people in the United States [3]. As of this writing, 15 different GSDs have been identified.

Myophosphorylase breaks the glycogen into glucose, and its deficiency causes glycogen accumulation in the skeletal muscle cells. This usually manifests in adolescence or early adulthood with muscle cramps and severe exercise intolerance. Diagnosis is suspected with laboratory findings of rhabdomyolysis and myoglobinuria, although genetic testing or a forearm muscle exercise test might also be needed [4-6].

This condition's exacerbation may be triggered by exercise, infection, or trauma [7]. This article presents a case of a young adult with COVID-19 with a history of McArdle disease who coursed with non-traumatic rhabdomyolysis and developed renal and liver failure.

## Case presentation

This is the case of a 27-year-old male with a past medical history of McArdle Disease (GSD type 4) and diabetes mellitus type 2 non-medicated, who presented to our emergency department with one week of muscle aches. The ache started on his upper arms and progressed towards the rest of his body. He described the symptoms as reminiscent of the last flares of his GSD; therefore, he tried hydration and rest, stated that his last crisis was more than three years ago. The day before he arrived at the emergency department, his symptoms worsened, with increased urinary frequency associated with increased fluid intake and dark-red urine. He also reported mild headaches, which resolved spontaneously, dry cough, and rhinorrhea. He reported contact with other SARS-CoV-2 patients and had only received one vaccine for COVID-19. He denied fever, shortness of breath, dysuria, dysgeusia, anosmia, lower extremity swelling, chest pain, abdominal pain, constipation, nausea, vomiting, diarrhea or active medication intake. 

On physical examination, he was alert, cooperative, and oriented. Hemodynamically stable, with an oxygen saturation of 100% on room air. The patient had a body mass index (BMI) of 54.8 kg/m². No pulmonary, cardiovascular, or skeletal abnormality or enlarged or swollen lymph nodes were noticed on the physical exam.

On admission, his laboratory analysis revealed a positive SARS-CoV-2 test, acute kidney disease, rhabdomyolysis, elevated liver enzymes, lactic acidosis, abnormal urine analysis, and negative toxicology (Table 1).

**Table 1:** Admission laboratory PCO2: Partial pressure of carbon dioxide, WBC: White blood cell, RBC: Red blood cell, ALT: Alanine transaminase, AST: Aspartate aminotransferase, Flu: Influenza, RSV: Respiratory syncytial virus, VB: Blood gas. *= Laboratory cannot report a specific value when creatinine kinase is above 40K.

BLOOD GASSES	Value		Reference
pH		7.36	7.35-7.45
PCO2		49.5	33-45 mmHg
Base Excess		2.70	-2 to +2 mEq/L
HCO3		28.1	23 - 30 mEq/L
O2 SAT		55.50	95 - 100%
Whole Blood Analysis			
Chloride, VB		101	95 - 105 mEq/L
Lactate		2.5	0.5 - 2.2 mmol/L
Glucose VB		152	70 - 100 mg/d
Potassium VB		3.2	3.5 - 5 mEq/L
Sodium VB		141	136 - 146 mEq/L
Ionized Calcium VB		1.17	1.20 - 1.40 mmol/L
Hematology			
WBC		7.3	4,500 - 11,000/mm3
RBC		6.12	4.35-5.65 mcL
Hemoglobin		16.5	13.5-17.5 g/dl
Hematocrit		49	41-50%
Platelet count		215	150,000-400,000/mm3
General chemistry			
Potassium, serum		3.3	3.5-5.5 mEq/L
Glucose, serum		134	70–100 mg/dL
Bun, serum		9	6 - 24 mg/dl
Creatinine, serum		1.20	0.6–1.2 mg/dL
Alkaline phosphatase, serum		68	25–100 U/L
Bilirubin		0.5	0.1–1.0 mg/dL
Direct Bilirubin		0.2	0.0–0.3 mg/dL
AST, SERUM		351	12–38 U/L
ALT, SERUM		93	10–40 U/L
C-REACTIVE PROTEIN		11.12	-10 mg/L
Creatinine kinase, serum		>3789	25–90 U/L
Toxicology		negative	
Virology			
SARS COV-2		Positive	
FLU A		Negative	
FLU B		Negative	
RSV		Negative	

Chest radiography showed perihilar interstitial prominence. He received potassium chloride and normal saline. He was not a candidate for monoclonal antibodies or steroids. On the third day of admission, routine labs showed inflammatory markers and high sensitivity d-dimer elevation. The lower extremity ultrasound was unremarkable for deep vein thrombosis. The patient also presented hyperglycemia of 217mg/dl (70-100mg/dl) and hyperkalemia of 5.3 mmol/L (3.5-5mmol/L) on suspicion that the elevated potassium was because of uncontrolled diabetes; the insulin sliding scale was started. Also, worsening transaminitis compatible with acute liver failure was observed (Table 2).

**Table 2:** Liver enzymes trending ALT: Alanine transaminase, AST: Aspartate aminotransferase

	Day 3	Day 4	Day 5	Day 6	Day 7	Day 8	Day 9	Day 10	Day 11	Day 12	Day 13	Day 14	Reference
AST	1349	1850	2122	1619	910	398	220	167	99	63	53	45	12-38 U/L
ALT	211	287	383	372	325	219	175	161	132	95	80	62	10-40 U/L

On the fourth day, his renal function worsened, given starting aggressive fluid therapy, an echocardiogram was done, and it revealed an ejection fraction of 62% with no signs of valvulopathy or vasculopathy. Despite the treatment for hyperglycemia, the potassium rose to 6.3 mmol/L (3.5-5 mmol/L); therefore, dextrose with regular insulin and sodium zirconium cyclosilicate was added to the therapy. 

Intravenous fluid was reinstated with a daily goal of 3-5 liters in spaced boluses. Creatinine plateaued with fluid management, and hypertension was managed with hydralazine as needed. On day four, chest radiography showed mild interstitial edema, intravenous fluids were held, and furosemide 20 mg oral was given once. Consequently, his renal function started to trend up (Table 3).

**Table 3:** Creatinine trending

	Day 3	Day 4	Day 5	Day 6	Day 7	Day 8	Day 9	Day 10	Day 11	Day 12	Day 13	Day 14	Day 15	Day 16	Reference
Creatinine	1.00	1.20	1.50	1.70	1.70	1.40	1.60	1.60	1.70	1.70	1.60	1.70	1.60	1.50	0.6 – 1.2 mg/dL

The patient showed clinical improvement and was ultimately discharged and followed as an outpatient without further complications. His new creatinine kinase (CK) baseline was 1.2-1.5 mg/dL.

## Discussion

GSD V, also known as McArdle disease, is caused by myophosphorylase deficiency [8]. An unusual condition is one of the most common metabolic myopathies [9]. During a crisis, the most common symptoms are fatigue and muscle aches [10].

Episodic rhabdomyolysis is a well-documented complication that could be present in up to half of the patients but rarely accounts for a severe form of the disease [11,12]. This periodical condition varies from asymptomatic elevated serum markers to potentially life-threatening. Numerous causes of rhabdomyolysis can be categorized into inherited or acquired and traumatic or non-traumatic. Several risk factors have been established, including toxins, metabolic disorders, infections, medications, seizures, and viral illnesses [13]. The rhabdomyolysis course can encompass various complications, including acute renal failure, electrolyte abnormalities, cardiac arrhythmias, liver dysfunction, and compartment syndrome [14]. Several reports have described rhabdomyolysis as a complication of viral infections, including COVID-19 [15]. The exact mechanisms are still unclear, but they are thought to be related to direct viral-mediated toxic effects on myocytes or elevation of myocyte toxic cytokines [16].

Severe rhabdomyolysis can cause a mild increase in liver enzymes. However, rare instances can associate it with fulminant acute liver failure. The incidence of hepatic dysfunction is up to 25% of patients with rhabdomyolysis [17,18]. Literature suggests that a disproportionate ratio of CK/aspartate aminotransferase (AST) and CK/alanine transaminase (ALT) could indicate hepatic injury in rhabdomyolysis [19,20].

Our literature review shows no documented cases of concurrent severe rhabdomyolysis and acute liver injury in a patient with McArdle disease with coexisting COVID-19 infection. Patients with COVID-19-induced rhabdomyolysis and those with McArdle disease can present similar symptoms, such as myalgias, tea-colored urine, and muscle weakness. Hence prompt recognition of possible complications is imperative.

Our patient had a disproportionate ratio of CK/AST, exhibiting liver injury in the setting of rhabdomyolysis and its well-known complication of acute kidney injury. This case report demonstrates that conservative treatment prevented a high-risk patient from undergoing renal replacement therapy. The prognosis in rhabdomyolysis is substantially worse if renal failure develops. However, in our case, a timely intervention with aggressive intravascular expansion was vital for complete renal and hepatic recovery.

## Conclusions

Myophosphorylase deficiency is a challenging disease to manage in the setting of SARS-CoV-2 infection. Clinical manifestations of both diseases can present similarly, such as myalgia and elevation of inflammatory markers, which make it difficult to differentiate between a flare-up of the GSD V or worsening SARS-CoV-2 infection. The prompt and accurate assessment is determinant to recognizing a life-threatening consequence of SARS-CoV-2, including complicated rhabdomyolysis, avoiding the patient's deterioration, and improving the outcome.
